# Supplementary material for: Network architecture of transcriptomic stress responses in zebrafish embryos
Source: PLoS Comput Biol. 2025 Jun 11;21(6):e1013164. doi: 10.1371/journal.pcbi.1013164 (PMC12184929; doi:10.1371/journal.pcbi.1013164)
Supplement: S1 File — (DOCX) [file pcbi.1013164.s002.docx]

Supplementary Material to the manuscript

Network architecture of the environmental stress response in zebrafish embryos

**Authors:**

Kaylee Beine^1^, Lauric Feugere^2^, Alexander P. Turner^3^, Katharina C. Wollenberg Valero^1,4^

**Author affiliations:**

^1^ School of Biology and Environmental Science, University College Dublin, Belfield, Dublin, Ireland

^2^ University of Quebec at Rimouski, Canada

^3^ Department of Computer Science, University of Nottingham, UK

^4^ Conway Institute, University College Dublin, Belfield, Dublin, Ireland

**Contains:**

**11 Supplementary Tables**

# **Supplementary Tables**

**Table A. Independent-sample Kruskal-Wallis test summary for differences between node types (interactome vs. genes with “stress response” GO term) for the three network statistical parameters.** The test statistic is adjusted for ties. ASPL - Average Shortest Path Length; NC - Neighborhood Connectivity; BC - Betweenness Centrality. Corresponding with Fig. 1B. Significant terms (p < 0.05) are shown in italics.

| **Network statistical parameter** | **Total N** | **Test Statistic**  **KW-H** | **Degree of Freedom** | **Asymptotic Sig. (2-sided test)** | **η^2^ [H]** |
| --- | --- | --- | --- | --- | --- |
| *ASPL* | *13791* | *122.454* | *1* | *<0.001* | *0.008* |
| NC | 13791 | 0.000 | 1 | 0.988 | *-0.00007* |
| *BC* | *11187* | *127.626* | *1* | *<0.001* | *0.011* |

**Table B. Independent-sample Kruskal-Wallis test summary for differences between node types (interactome vs. experimental DEGs) for the three network statistical parameters.** The test statistic is adjusted for ties. ASPL - Average Shortest Path Length; NC - Neighborhood Connectivity; BC - Betweenness Centrality. Corresponding with Fig. 3 (A-C). Significant terms (p < 0.05) are shown in italics.

| **Network statistical parameter** | **Total N** | **Test Statistic**  **KW-H** | **Degree of Freedom** | **Asymptotic Sig. (2-sided test)** | **η^2^ [H]** |
| --- | --- | --- | --- | --- | --- |
| *ASPL* | *14360* | *20.778* | *3* | *<0.001* | *0.001* |
| *NC* | *14360* | *19.837* | *3* | *<0.001* | *0.001* |
| *BC* | *11658* | *19.795* | *3* | *<0.001* | *0.001* |

**Table C. ASPL Pairwise comparisons from Mann-Whitney Wilcoxon tests.** Tests for difference in average shortest path length between DEGs from the different treatment comparisons against the interactome. All significance values have been adjusted by Bonferroni correction for multiple tests. Corresponding with Fig. 3A. Significant terms (p < 0.05) are shown in italics.

| **Gene set 1** | **Gene set 2** | **W** | **Std. Error** | **Std. W** | **P** | ***p-*adj.** |
| --- | --- | --- | --- | --- | --- | --- |
| interactome | TS | -217.451 | 199.739 | -1.089 | 0.276 | 1 |
| *interactome* | *UV* | *420.675* | *99.165* | *4.242* | *0* | *0* |
| interactome | TS->UV | -88.624 | 167.256 | -0.53 | 0.596 | 1 |

**Table D. NC Pairwise comparison from Mann-Whitney Wilcoxon tests.** Tests for difference in neighborhood connectivity between DEGs from the different treatment comparisons against the interactome. All significance values have been adjusted by Bonferroni correction for multiple tests. Corresponding with Fig. 3B. Significant terms (p < 0.05) are shown in italics.

| **Gene set 1** | **Gene set 2** | **W** | **Std. Error** | **Std. W** | **P** | ***p-*adj.** |
| --- | --- | --- | --- | --- | --- | --- |
| *interactome* | *TS* | *630.619* | *199.739* | *3.157* | *0.002* | *0.01* |
| interactome | UV | -130.705 | 99.165 | -1.318 | 0.187 | 1 |
| *interactome* | *TS->UV* | *453.921* | *167.256* | *2.714* | *0.007* | *0.04* |

**Table E. BC Pairwise comparison from Mann-Whitney Wilcoxon tests.** Tests for difference in betweenness centrality between DEGs from the different treatment comparisons against the interactome. All significance values have been adjusted by Bonferroni correction for multiple tests. Corresponding with Fig. 3C. Significant terms (p < 0.05) are shown in italics.

| **Gene set 1** | **Gene set 2** | **W** | **Std. Error** | **Std. W** | ***p*** | ***p-*adj.** |
| --- | --- | --- | --- | --- | --- | --- |
| *interactome* | *TS* | *-525.452* | *178.755* | *-2.94* | *0.003* | *0.02* |
| interactome | UV | -158.669 | 87.9 | -1.805 | 0.071 | 0.426 |
| *interactome* | *TS->UV* | *-481.9* | *150.028* | *-3.212* | *0.001* | *0.008* |

**Table F. Independent-sample Kruskal-Wallis test summary for differences between node types (expressed network vs. experimental DEGs) for the three network statistical parameters.** The test statistic is adjusted for ties. ASPL - Average Shortest Path Length; NC - Neighborhood Connectivity; BC - Betweenness Centrality. Corresponding with Fig 4 (A-C). Significant terms (p < 0.05) are shown in italics.

| **Network statistical parameter** | **Total N** | **Test Statistic**  **KW-H** | **Degree of Freedom** | **Asymptotic Sig. (2-sided test)** | **η^2^ [H]** |
| --- | --- | --- | --- | --- | --- |
| *ASPL* | *11321* | *13.226* | *3* | *0.004* | *0.0009* |
| *NC* | *11321* | *20.253* | *3* | *<0.001* | *0.002* |
| BC | 9395 | 6.297 | 3 | 0.098 | *0.0004* |

**Table G. ASPL Pairwise comparisons from Mann-Whitney Wilcoxon tests.** Tests for difference in average shortest path length between DEGs from the different treatment comparisons against the expressed network. Corresponding with Fig. 4A. Significant terms (p < 0.05) are shown in italics.

| **Gene set 1** | **Gene set 2** | **W** | **Std. Error** | **Std. W** | ***p*** | ***p-*adj.** |
| --- | --- | --- | --- | --- | --- | --- |
| Expressed network | TS | -341.475 | 158.596 | -2.153 | 0.031 | 0.188 |
| Expressed network | UV | 154.906 | 80.423 | 1.926 | 0.054 | 0.325 |
| Expressed network | TS->UV | -246.017 | 133.203 | -1.847 | 0.065 | 0.389 |

**Table H. NC Pairwise comparisons from Mann-Whitney Wilcoxon tests.** Tests for difference in neighborhood connectivity between DEGs from the different treatment comparisons against the expressed network. Corresponding with Fig. 4B. Significant terms (p < 0.05) are shown in italics.

| **Gene set 1** | **Gene set 2** | **W** | **Std. Error** | **Std. W** | ***p*** | ***p-*adj.** |
| --- | --- | --- | --- | --- | --- | --- |
| *Expressed network* | *TS* | *504.88* | *158.595* | *3.183* | *0.001* | *0.009* |
| Expressed network | UV | -103.597 | 80.423 | -1.288 | 0.198 | 1 |
| *Expressed network* | *TS->UV* | *362.267* | *133.203* | *2.72* | *0.007* | *0.039* |

**Table I.** Antagonistic and synergistic independent-sample Kruskal-Wallis test summary for differences between node types (interactome vs. antagonistic/synergistic) for the three statistical parameters. The test statistic is adjusted for ties. ASPL - Average Shortest Path Length; NC - Neighborhood Connectivity; BC - Betweenness Centrality. The significance level is .050. Corresponding with Fig. 6 (B and D). Significant terms (p < 0.05) are shown in italics.

| **Network statistical parameter** | **Total N** | **Test Statistic**  **KW-H** | **Degree of Freedom** | **Asymptotic Sig. (2-sided test)** | **η^2^ [H]** |
| --- | --- | --- | --- | --- | --- |
| **Antagonistic** | | | | | |
| ASPL | 13791 | .000 | 1 | .991 | -7.25E-05 |
| NC | 13791 | 1.340 | 1 | .247 | 2.47E-05 |
| BC | 13791 | .010 | 1 | .922 | -7.18E-05 |
| **Synergistic** | | | | | |
| ASPL | 13791 | 1.235 | 1 | .266 | 1.70E-05 |
| NC | 13791 | 3.129 | 1 | .077 | 0.0002 |
| BC | 13791 | .297 | 1 | .586 | -5.10E-05 |

**Table J.** Thermal stress and UV exposure as single stressors independent-sample Kruskal-Wallis test summary for differences between node types (interactome vs. single stressors) for the three statistical parameters. The test statistic is adjusted for ties. ASPL - Average Shortest Path Length; NC - Neighborhood Connectivity; BC - Betweenness Centrality. The significance level is .050. Corresponding with Fig. 6 (B and D). Significant terms (p < 0.05) are shown in italics.

| **Network statistical parameter** | **Total N** | **Test Statistic**  **KW-H** | **Degree of Freedom** | **Asymptotic Sig. (2-sided test)** | **η^2^ [H]** |
| --- | --- | --- | --- | --- | --- |
| **TS single stressor** | | | | | |
| ASPL | 2599 | 2.828 | 1 | .093 | 0.0007 |
| NC | 2599 | 1.780 | 1 | .182 | 0.0003 |
| BC | 2599 | .333 | 1 | .564 | -0.0002 |
| **UV single stressor** | | | | | |
| *ASPL* | *2599* | *12.416* | *1* | *<.001* | *0.004* |
| *NC* | *2599* | *16.584* | *1* | *<.001* | *0.006* |
| BC | 2599 | .207 | 1 | .649 | -0.0003 |

**Table K. Pie charts in Fig 2-6.** Numbers and percentages of nodes per category in different networks or gene sets, and their change in comparisons.

| **Comparison** | **Figure #** | **Experimental comparison group** | **Percentage of targeted H nodes, Total H node percentage,**  **Difference in number of nodes** | **Percentage of targeted I nodes, Total I node percentage,**  **Difference in number of nodes** | **Percentage of targeted P nodes, Total P node percentage,**  **Difference in number of nodes** |
| --- | --- | --- | --- | --- | --- |
| **Interactome** | **Fig. 1**  **Fig. 3**  **Fig. 5**  **Fig. S1** |  | 2.13%, | 35.7%, | 62.1%, |
| **stress GO term genes vs. interactome** | **Fig. 1** | Stress response (GO term)  N= 1176 | 4.19%,  +2.06%,  +24 | 37%,  +1.3%,  +15 | 58.9%,  -3.2%,  -38 |
| **Experimental DEGs vs interactome** | **Fig. 3** | Thermal stress  N=461 | 3.79%,  +1.33%,  +6 | 29.7%,  -6%,  -27 | 66.5%,  +4.4%,  +20 |
|  |  | UV exposure  N=2113 | 2.41%,  +0.28%,  +6 | 37.6%,  +1.9%,  +40 | 60%,  -2.1%,  -44 |
|  |  | UV preceded by thermal stress  N=666 | 2.46%,  +0.33%,  +2 | 33.2%,  -2.5%,  -17 | 64.3%,  +2.2%,  +15 |
| **Expressed network** | **Fig. 4** |  | 0.25% | 74% | 25.7% |
|  |  | Expressed network vs interactome | -1.88%, | +38.3%, | -36.4%, |
| **Experimental DEGs vs. expressed network** | **Fig. 4** | Thermal stress  N=461 | 0.445%,  +0.2%,  + 1 | 66.8%,  -7.2%,  -33 | 32.7%,  +7.07%,  +33 |
|  |  | UV exposure  N=2113 | 0.386%,  +0.059%,  +1 | 75%,  +1%,  +21 | 24.6%,  -1.1%,  -23 |
|  |  | UV preceded by thermal stress  N=666 | 0%,  -0.25%,  -2 | 70.7%,  -3.3%,  -22 | 29.3%,  +3.6%,  +24 |
| **Stressor-unique DEGs and interactome** | **Fig. 5** | TS single stressor  N=134 | 3.05%,  +0.92%,  +1 | 29%,  -6.7%,  -9 | 67.9%,  +5.8%,  +8 |
|  |  | UV single stressor  N=1720 | 2.21%,  -0.08%,  0 | 38.4%,  +2.7%,  +5 | 59.4%,  -2.7%,  -5 |
| **Antagonistic and synergistic genes vs interactome** | **Fig. S1** | Antagonistic  N=135 | 4.17%,  +2.04%,  +2.8 | 27.8%,  -7.9%,  -11 | 68.1%,  +6%,  +8 |
|  |  | Synergistic  N=287 | 2.05%,  -0.08%,  0 | 32.2%,  -3.5%,  -10 | 65.8%,  +3.7%,  +11 |
