# Supplementary material for: Network architecture of transcriptomic stress responses in zebrafish embryos
Source: PLoS Comput Biol. 2025 Jun 11;21(6):e1013164. doi: 10.1371/journal.pcbi.1013164 (PMC12184929; doi:10.1371/journal.pcbi.1013164)
Supplement: Attachment — Submitted filename: Response_to_reviewers.docx [file pcbi.1013164.s005.docx]

We would like to thank the reviewer for their positive evaluation of our manuscript. Below, we respond to each criticism and detail which changes we have made to the manuscript to enhance readability and provide more details.

Best wishes

On behalf of all authors,

Katharina Valero

**Reviewers' comments:**

**Reviewer #1: Beine et al. examined the topology of stress response networks in zebrafish embryos. They utilized transcriptional data to find experimental evidence for network conservation in response to stressors. They proposed and tested four hypotheses through analyses such as network comparison and pathway enrichment. Overall, the study is compelling, and the findings are significant. However, the manuscript would benefit from clearer explanations of the results and a more concise, precise summary of the key findings in the abstract.**

We thank the Reviewer for their positive feeling about our study and its significance. We provide them with an improved, revised version, where we made the changes they suggested below. We are confident this revision strengthens our manuscript and provides clearer, more concise conclusions. Particularly, in the abstract, we have made the following changes:

We have added in more concise language such as “and its associated genes highly expressed” (page 2, lines 21-22); indicated the UV “transcripts” (page 2, line 27) and changed the order of the sentences, placing the results found in the last few sentences of the abstract (page 2, lines 29 - 32), keeping the word limit consistent.

**More detailed comments are as follows:**

**The full names of terms should be introduced before using their acronyms, such as ASPL, BC, NC and KW-H. An explanation of how each of these metrics contributes to node centrality should be included.**

This was included in the methods but has now been included in the introduction for clarity and flow of reading. Authors have included the acronyms and an explanation on the network statistics for ease of reading in the introduction (pages 5-6, lines 124-136).

*“Networks were classified based on three characteristics to show and derive meaning from the positioning of proteins within the PPI network: Neighborhood Connectivity (NC, with the highest values characterized nodes located intermediately in the network and with the highest number of edges to other nodes), Average Shortest Path Length (ASPL, with the highest values characterizing nodes peripherally located in the network and with few connections to other nodes), and Betweenness Centrality (BC, with highest values characterizing nodes central to the network) [34]****.*”**

**Figure 3, 4, 5 demonstrate that stressors employ distinct strategies to achieve centrality. For example, in Figure 3, TS had similar ASPL but a lower NC and higher BC. UV had a significantly lower ASPL and no significant difference in NC and BC. The authors should include an explanation or deeper exploration on these different network organization strategies.**

We thank the Reviewer for this thoughtful observation. We agree that the distinct patterns of node usage across stress treatments merit further clarification. However, we would like to note that “centrality” as a unidimensional concept is not the most accurate descriptor within our framework. Instead, the categories of H, I, and P nodes represent distinct topological shells within a three-dimensional network space. While traditional network layouts often show dense clusters near the center of a graph, topological density does not necessarily correspond to visual centrality. In our framework, we use a three-shell model, analogous to the internal structure of the Earth:

- (i) H nodes (high betweenness centrality) represent the core, connecting distant modules and facilitating cross-network integration.
- (ii) I nodes (high neighborhood connectivity) form the outer core, acting as the main hubs of internal communication.
- (iii) P nodes (high average shortest path length) occupy the crust, reflecting more isolated, specialized, and loosely connected nodes.

This framework allows us to interpret stressor effects in terms of how deeply embedded their transcriptomic responses are within the organism’s systemic network.

Building on this, we now offer a more detailed interpretation of the distinct stressor-specific patterns observed in Figures 3, 4, and 5:

- (i) TS (thermal stress) and TS->UV (sequential stress) DEGs exhibited low neighborhood connectivity (NC) but high betweenness centrality (BC), suggesting a strategy focused on cross-module integration. These stressors appear to activate central genes that act as bridges between otherwise disconnected functional regions, potentially coordinating broader systemic responses.
- (ii) In contrast, UV-responsive DEGs showed significantly lower ASPL but no significant differences in NC or BC, implying a globally and locally clustered response within tightly connected subnetworks—consistent with the modular activation of DNA repair and apoptosis pathways, as characterized in our functional enrichment analyses.

We have now added a paragraph to the Discussion section (pages 21-22, lines 456-469) to explicitly interpret these distinct topological strategies and clarify how network structure informs the systemic stress response:

*“Our results indicate that different environmental stressors engage with network architecture through distinct topological strategies. Thermal stress (TS) and the combined treatment (TS->UV) activated genes with high betweenness centrality but lower connectivity, suggesting a bridging role across central modules. This implies a more integrative response, where select genes coordinate communication between functionally distinct regions of the transcriptome. In contrast, UV-responsive genes exhibited significantly lower average shortest path lengths but no significant differences in neighborhood connectivity or betweenness centrality. This pattern is indicative of both global and modular, internally cohesive activation, which could potentially reflect targeted engagement of tightly connected pathways related to DNA repair and apoptosis. These contrasting strategies - cross-module integration versus modular activation - highlight how transcriptomic stress responses are not only shaped by the nature of the stressor, but also by the underlying network topology through which they are enacted.*”

**The conclusion, "Combined stressors activate more central nodes while stressor-unique DEGs are more peripheral," is not clearly supported by the results. While the text mentions a slight increase in H nodes for combined stressors, no significant differences in ASPL, BC, or NC are reported. Statistical metrics quantifying these centrality changes should be included to substantiate the findings. For UV single stressors, the activation of more I nodes and fewer P nodes, along with lower ASPL and BC, is noted. However, the authors should clarify the criteria for defining these changes as “more peripheral” and ensure their significance is adequately supported.**

The authors agree with the reviewer’s assessment that we cannot say “Combined stressors activate more central nodes while stressor unique DEGs are more peripheral” as this is not backed up significantly. This was clarified by changing the sentence to “Combined stressors show unstructured positioning while stressor-unique DEGs show centralization within the network.” The discussion was refined, and conclusions were reformed to reflect the centralization of the network by single stressors (specifically UV). Research suggests that due to UV being a mutagen, it affects genes that are essential to life and this is therefore reflected in the DEGs unique to UV showing significant centralization within the network.

**The authors described the co-variation of DEG network parameters and phenotypic outcomes in lines 260–267. Adding a discussion on these results would provide clearer insights into how network topology/metrics influences phenotypic outcomes.**

We thank the Reviewer for this suggestion. We have now added a paragraph to the Discussion to explicitly address the relationship between network topology and phenotypic outcomes, clarifying how variation in ASPL, NC, and BC across treatments relates to defect rates and embryo length. We have added a sentence to the Discussion (pages 22-23, lines 484-491), also connecting these results with those related to fold change in fig 8.

*“Low defect scores and greater embryo length in TS and TS->UV corresponded with low ASPL and high NC, while UV-treated embryos showed lower BC and increased defects. This suggests that both modular cohesion (via NC, in intermediate network positions) and cross-module integration (via BC, in central positions) contribute to mitigating stress effects. These trends align with our fold change analysis, where genes with high pleiotropic impact - mostly I or H nodes - exhibited lower expression shifts but were more phenotypically influential. Together, these results highlight the role of network topology in modulating phenotypic resilience during development.”*

**In Figures 1, 2-4, the axes for ASPL, NC, and BC should have consistent scales across to avoid confusion. For example, in the middle panel of Figure 1B, the difference in NC appears dramatic, but the statistical test indicates it is insignificant. The y-axis scale in the left and right panels also exaggerate the differences in ASPL and BC, making them appear more significant than they are. Also, there is an error in the text – “higher but not significantly different NC”, which is not consistent with the result in the figure.**

The text meant to read “lower but not significantly different NC” (page 8, line 196-197) and has been changed accordingly. All error plots have been changed to now have a consistent range throughout to facilitate comparisons: Average shortest path length 3.95 – 4.30; neighbourhood connectivity 60 – 85; betweenness centrality 0 – 0e-03 (0.001)

**In Figure 2B, the Venn diagram should represent the size of different DEG numbers proportionally to provide a more accurate visual representation of their relative sizes.**

The venn diagram (Figure 2) has been changed to a proportional venn diagram to reflect the different DEG numbers visually.

**In figure 5A, overlay the yellow dots on top to better compare the distributions.**

The authors do agree that overlaying the dots will show better in the DFA, however the dots are in a 3D spatial model, if they are overlaid, their positioning is compromised. Therefore, this figure was not changed.

**Figure 7 misses color legend for green, light green, dark green, and turquoise, making it difficult to identify the colors from their names accurately.**

A legend has been added for the shades of green and turquoise for more clarity.

**Avoid using informal language such as “usually,” “mostly,” and “some” when describing scientific results (e.g., lines 28–29, line 42).**

Authors removed “usually”, “mostly” and “some” from manuscript

**Line 132, Protein-Protein Interaction (PPI) → PPI**

Authors removed “Protein-Protein Interaction” and left PPI

**Line 264：SEL is the sum of embryo length? “ASPL NC” —> “ASPL and NC”**

SEL stands for “shortest embryo length”. Accordingly, the authors added “…shortest embryo length (SEL)…” (page 15, line 339) and “and” between ASPL and NC

**Have the authors made all data and (if applicable) computational code underlying the findings in their manuscript fully available?**

All downloaded data and computational codes underlying the finding in this paper have been added: https://github.com/KBeine/Network_architecture_zebrafish_PPI/tree/main

**The PLOS Data policy requires authors to make all data and code underlying the findings described in their manuscript fully available without restriction, with rare exception (please refer to the Data Availability Statement in the manuscript PDF file). The data and code should be provided as part of the manuscript or its supporting information, or deposited to a public repository. For example, in addition to summary statistics, the data points behind means, medians and variance measures should be available. If there are restrictions on publicly sharing data or code —e.g. participant privacy or use of data from a third party—those must be specified.**

Raw data was supplied in a supplementary dataset and script:

1 Supplementary Dataset - Supplementary dataset.xlsx

1 Supplementary Script – <https://github.com/KBeine/Network_architecture_zebrafish_PPI/tree/main>
